# Evaluation of biases in remote photoplethysmography methods

**DOI:** 10.1038/s41746-021-00462-z

**Published:** 2021-06-03

**Authors:** Ananyananda Dasari, Sakthi Kumar Arul Prakash, László A. Jeni, Conrad S. Tucker

**Affiliations:** 1 Department of Mechanical Engineering, Carnegie Mellon University, Pittsburgh, PA USA; 2 The Robotics Institute, Carnegie Mellon University, Pittsburgh, PA USA; 3 Machine Learning Department, Carnegie Mellon University, Pittsburgh, PA USA; 4 Department of Biomedical Engineering, Carnegie Mellon University, Pittsburgh, PA USA; 5 CyLab Security and Privacy Institute, Carnegie Mellon University, Pittsburgh, PA USA

**Keywords:** Biomarkers, Developing world

## Abstract

This work investigates the estimation biases of remote photoplethysmography (rPPG) methods for pulse rate measurement across diverse demographics. Advances in photoplethysmography (PPG) and rPPG methods have enabled the development of contact and noncontact approaches for continuous monitoring and collection of patient health data. The contagious nature of viruses such as COVID-19 warrants noncontact methods for physiological signal estimation. However, these approaches are subject to estimation biases due to variations in environmental conditions and subject demographics. The performance of contact-based wearable sensors has been evaluated, using off-the-shelf devices across demographics. However, the measurement uncertainty of rPPG methods that estimate pulse rate has not been sufficiently tested across diverse demographic populations or environments. Quantifying the efficacy of rPPG methods in real-world conditions is critical in determining their potential viability as health monitoring solutions. Currently, publicly available face datasets accompanied by physiological measurements are typically captured in controlled laboratory settings, lacking diversity in subject skin tones, age, and cultural artifacts (e.g, bindi worn by Indian women). In this study, we collect pulse rate and facial video data from human subjects in India and Sierra Leone, in order to quantify the uncertainty in noncontact pulse rate estimation methods. The video data are used to estimate pulse rate using state-of-the-art rPPG camera-based methods, and compared against ground truth measurements captured using an FDA-approved contact-based pulse rate measurement device. Our study reveals that rPPG methods exhibit similar biases when compared with a contact-based device across demographic groups and environmental conditions. The mean difference between pulse rates measured by rPPG methods and the ground truth is found to be ~2% (1 beats per minute (b.p.m.)), signifying agreement of rPPG methods with the ground truth. We also find that rPPG methods show pulse rate variability of ~15% (11 b.p.m.), as compared to the ground truth. We investigate factors impacting rPPG methods and discuss solutions aimed at mitigating variance.

## Introduction

Changes in physiological signals of the human body, such as pulse rate, body temperature, blood pressure, and respiration rate can be monitored using invasive (sensors are inserted into the body) or noninvasive (sensors are not inserted into the body) methods^1^. Noninvasive methods are further classified as contact (sensor makes contact with the skin) and noncontact (take measurements from a distance) methods^2^. Contact-based methods monitor physiological signals by measuring changes in physical properties, such as pressure, temperature, and light transmitted/reflected^3^. Noncontact physiological signal monitoring is primarily achieved using camera, audio, infrared (IR), ultrasound, or Doppler-based approaches (Advanced Non-contact Patient Monitoring Technologies: A New Paradigm in Healthcare Monitoring), each differing in the type of input signal used for measurement. These approaches have gained momentum for remote monitoring of physiological signals of subjects (Advanced Non-contact Patient Monitoring Technologies: A New Paradigm in Healthcare Monitoring). Of these methods, video modality has become the predominant input data type for noncontact approaches, driven by the availability of low-cost cameras and smartphones^4^. The COVID-19 pandemic has also contributed to increased usage of noncontact technology for monitoring vital signs of patients, as well as analyzing the effects of new drugs^5^. Remote photoplethysmography (rPPG) is a noncontact video-based method that monitors the change in blood volume by capturing pixel intensity changes from the skin to measure pulse rate^6^. In contrast, contact-based photoplethysmography (PPG) sensors emit light onto the skin and measure the pulse rate by detecting the amount of light transmitted or reflected (i.e., Skin reflection model^7^). PPG approaches may not be suitable for vital sign measurements in situations involving contagious diseases (e.g., COVID-19), due to the need for physical contact and sterilization procedures after use^8^. rPPG approaches make no physical contact with the person whose physiological measurements are being captured, making them suitable for situations necessitating noncontact approaches. Moreover, rPPG methods have the potential to be integrated into existing mobile phones (e.g., via an app download), especially given the increased prevalence of mobile devices in resource constrained environments^4^.

The differences between estimated and actual physiological signals can be due to multiple sources of noise^9,10^. Sensor noise varies with the type of sensor, conditions during measurement, human error, and bias due to subject demographics^9^. The measurement noise for PPG methods across subject demographics has been studied in detail by Bent et al.^11^. The quantification of rPPG measurement biases across demographics remains an open area of scientific inquiry. The primary objective of this work is to investigate sources of error across state-of-the-art rPPG methods and an FDA-approved gold standard pulse rate measurement device in demographic populations typically not represented in evaluation datasets. We investigate factors responsible for causing pulse rate estimation bias, while using state-of-the-art rPPG methods on facial videos.

**Fig. 1:**
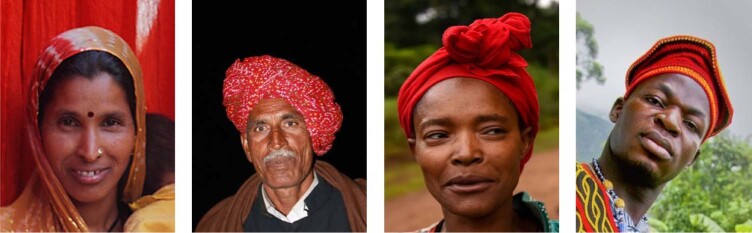
Representative images illustrating challenging facial features (“bindi”, headgear). The images from left to right are ‘nestle, jodhpur’ by ‘nevil zaveri’ licensed under CC BY 2.0, ‘Sawai Madhopur, man with turban’ by ‘Arian Zwegers’ licensed under CC BY 2.0, ‘Wollayta Woman’ by ‘Rod Waddington’ licensed under CC BY-SA 2.0, and ‘Raymond’ by ‘Michael Downey’ licensed under CC BY 2.0.

Commonly used datasets for tasks related to processing facial videos, such as BP4D and Multi-Pie, are unbalanced in terms of demographic diversity^12,13^. A majority of subjects are from Euro-American (49%) descent followed by Asians (27%) in the age group of 19–29 years. Other publicly available datasets, such as MAHNOB-HCI and MMSE-HR, also show similar demographic composition^14^. These datasets are also collected under optimal illumination conditions in controlled laboratory settings. In contrast, the dataset used for this study is collected from two geographically and culturally distinct countries, India and Sierra Leone, introducing environmental and subject variations that are typically underrepresented in publicly available face datasets. Increasing the diversity of datasets is critical to minimizing algorithmic biases. Further, the demographic representation in the dataset used in this study contains subjects with features, such as facial marks or adornments and headgear (Fig. 1), which are typically not represented in existing datasets used to train or evaluate rPPG methods. For example, the presence of facial marks, such as a “bindi” worn by Indian women, may interfere with spatial pixel averaging in the forehead skin region of interest and may cause biased estimation of pulse rate. The presence of headgear, such as hats or turbans, may cause occlusion leading to errors in face detection. A knowledge gap exists in terms of how well existing state-of-the-art rPPG methods perform when presented with diverse datasets. This work

- Performs data collection of facial videos of subjects containing demographics typically not represented in existing datasets used to evaluate rPPG methods for pulse rate estimation.
- Compares and tests the estimation accuracy of state-of-the-art rPPG methods against an FDA-approved ground truth PPG sensor on the collected demographically diverse dataset.
- Investigates the sources of bias in rPPG approaches for pulse rate estimation, such as country of origin, gender, and skin tone, and quantifies the error due to each identified source.

Prior work on PPG-based pulse rate measurement methods can be classified into PPG and rPPG methods. rPPG methods are divided into traditional image processing approaches and deep-learning approaches, depending on the type of algorithm used. The following sections discuss studies on PPG and rPPG sensor development, signal processing, and algorithm testing, highlighting the contributions of the current work.

### Contact-based PPG

Research in the area of contact-based PPG by Yang et al. and Rhee et al. started with the development of wireless finger ring-based sensors for continuous monitoring of heart rate. These studies used the red and IR components of transmitted light to monitor heart rate over an extended period of time^15,16^. Renevey et al. developed a wrist-based sensor using reflected IR light to measure pulse rate^17^. Further, the study proposes automatic noise cancellation methods to filter the input signal. Mendelson and Pujary studied the effect of site of pulse rate measurement on the readings for a wrist and forehead-based sensor^18^. Wang et al. and Celka et al. developed sensors that can be placed on the ear to measure pulse rate^19,20^. Celka et al. also introduced a principal component analysis (PCA)-based noise reduction method that reduces disturbances due to the subject motion^20^. Ear-based sensors were further modified, and embedded in earrings and earphones in studies by Poh et al.^21^. The main focus of the aforementioned studies was the development of a physical sensor and the identification of reliable measurement locations on a human body for pulse rate, such as the wrist, forehead, and ear. They also included analysis of noise reduction and signal processing methods to extract high-quality PPG signals. These studies also focused primarily on the IR and red regions of the spectrum as studies^22^ showed that these regions contain useful PPG information. The studies however did not include a discussion of the accuracy or measurement bias of the wrist, forehead, and ear-based sensors due to environmental or demographic variations in the subjects tested. Work by Tamura details the principles of transmission and reflection-based PPG methods^23^. The study finds the green channel to be suitable for reducing errors due to small body movements. The findings were based on experiments conducted on a single individual, using different sites for video-based pulse rate measurement. A study by Tautan explores the correlation of PPG signals to different types of motion and wavelength of light, using accelerometer data for validation^24^. The designed algorithm improves the visibility of the heart rate component in the frequency domain of the signal. The study however performs validation on a small population and gives no quantification of the measurement bias due to demographic diversity in the subjects. The development of PPG devices has been studied in detail by Castaneda^25^. The study discusses the types of PPG sensors, the possible sites of measurement, and sources of error. An analysis of the accuracy of the PPG method from diverse populations typically not represented in evaluation datasets is not conducted. Zhang developed an algorithmic framework to reduce the error in detected pulse rate due to wrist motion in a contact-based wrist PPG sensor^26^. The study succeeds in reducing the error due to small wrist movements by using an IR PPG sensor as the motion reference. Generalization to larger movements and different subject and environmental variations has not been performed. Recent work in the area of contact-based PPG by Bent et al. investigates the accuracy of wearable device measurements obtained from PPG sensors from multiple manufacturers^11^. The study compares the accuracy of the sensor measurements with the ground truth pulse readings from an electrocardiogram (ECG), across subjects of varying skin tones. It also includes an analysis of errors due to the sensor movement and physical activity, as well as variations among different manufacturers. The study analyzes measurement bias of contact-based sensors in measuring the pulse rate of both stationary and exercising subjects in a laboratory setting.

Contact-based PPG algorithms performed tests on subject groups with a largely homogeneous demographic distribution (Caucasians in the age range of 19–29). Moreover, the subjects were tested in stationary laboratory settings, which is not representative of outside-the-lab conditions, that typically include subject and background motion, as well as nonuniform illumination changes. The current work develops a dataset that is collected from diverse subjects in their representative environments. An FDA-approved ground truth PPG and state-of-the-art rPPG approaches are evaluated on this dataset.

### Image processing approaches for remote PPG

Early work in the area of rPPG was conducted by Verkruysse et al. in the identification of the correct channel of ambient light for best results^27^. The study shows that though the green channel contains the most useful PPG information, the red and blue channels contain important PPG information as well. Work by Lewandowska et al. and Poh et al. used feature selection algorithms (PCA and independent component analysis (ICA), respectively) to extract useful features to obtain the desired PPG signal^28,29^. Work on Eulerian video magnification by Hao-Yu Wu et al. demonstrated a spatial decomposition and temporal filtering approach to visualize small temporal changes (caused by blood flow) in facial videos, leading to better detection of pulse rate^30^. The algorithm was evaluated on three individuals with different skin tones with a ground truth ECG. Work by De Haan and Van Leest used the difference in absorption spectra of bloodless skin and arterial blood to design a more “motion-robust”, chrominance-based remote PPG algorithm (CHROM)^31^. This algorithm was compared to a range of contact-based fitness devices, and was found to be of comparable accuracy for both stationary and moving subjects. The subject videos were captured in laboratory conditions, as opposed to the dataset we developed, where subject videos are captured in participants’ natural environments that include artifacts typically not found in laboratory-controlled environments. The Plane Orthogonal-to-Skin (POS) algorithm developed by Wang et al., projects the PPG signal on to a plane orthogonal to the skin tone to extract the pulse signal. The algorithm was tested on subjects with different skin tones and with different activity levels, in a laboratory setting and was found to outperform CHROM and PCA/ICA algorithms^32^. Wang et al. also developed a “Spatial Subspace Rotation” (SSR) algorithm that observes a subspace of skin pixels over time and measures their “rotation” for pulse extraction^33^. Subjects with varying skin tones and under different illumination and activity conditions were tested under laboratory conditions. The SSR algorithm outperforms previous source separation methods (ICA) and CHROM. Partial demographic analysis (across skin tones) was performed in this study. The VideoVitals approach developed by Prakash and Tucker employs a bounded Kalman Filter (BKF) and a denoising algorithm to reduce the effect of motion and improve feature tracking^34^. The BKF algorithm is tested on subjects performing a range of head motions and is shown to outperform the existing methods for reducing motion inaccuracies. Similar to other state-of-the-art methods, the performance of BKF was tested on subjects from a university setting, under laboratory conditions. The Spherical Mean approach developed by Pilz et al. describes a lower-dimensional representation of pixel intensities, using a geodesic sphere. This representation unifies the invariance properties with respect to a translation of features, increasing the robustness to head motion and also eliminating the need for parameter tuning^35^.

Image processing-based approaches for rPPG have been shown to perform better than contact-based sensors for pulse rate determination. The need for physical contact is minimized, increasing the versatility of these rPPG methods. Image processing approaches often involve multiple steps, such as face tracking, skin segmentation, color space transformation, feature selection, and noise filtering. Deep-learning approaches minimize the requirement for multiple stages of processing and also automate the task of feature selection.

### Deep-learning approaches for remote PPG

The automation of feature selection and the reduction of multistep video processing warrants the use of deep-learning-based approaches for pulse rate estimation. “DeepPhys” is a deep-learning-based convolutional attention network developed by Chen and McDuff, that estimates the PPG signal from an input facial video, using a finger pulse oximeter signal as the reference pulse^36^. The model consists of two subnetworks, the first to identify the skin region of interest in the facial video and the second to estimate the PPG signal from the frame difference in the observed frames. The method was tested on subjects with a demographic (age, gender, and skin tone) composition similar to the dataset we collected, but in laboratory settings with a synthetic background. SynRhythm, developed by Niu et al., is a transfer learning approach, where spatiotemporal features are directly converted to heart rate^37^. This is tested on public domain video datasets, which feature mainly Caucasian subjects in a static setting (MAHNOB-HCI and MMSE-HR). HR-CNN is another model developed by Speltik et al. using two convolutional neural networks with different loss functions, to first extract face regions of interest and use the extracted signals to predict heart rate^38^. This model was tested on public domain datasets and on subjects with high activity levels. PhysNet, developed by Yu et al. uses a 3D-CNN followed by an RNN to learn spatiotemporal facial features and extract the PPG signal^39^. Testing on public domain datasets shows improved correlation with the ground truth (ECG) and improved performance over non deep-learning methods, such as CHROM and POS. Deep-learning methods have been shown to perform better than traditional image processing-based approaches in pulse rate determination from a facial video. Current state-of-the-art deep-learning models train using large scale, diverse training datasets^40–44^, and complex architectures such as attention masks^45–48^. While these approaches have resulted in highly accurate models^49–51^, there is a risk of overfitting to the training data. Deep-learning-based rPPG networks were tested on subjects from primarily Caucasian backgrounds and in laboratory conditions. The performance of deep-learning algorithms on subjects with diverse demographic backgrounds and for videos captured outside-the-lab, has not been evaluated.

In this work, we evaluate state-of-the-art rPPG algorithms on subjects from different demographic groups in their natural environmental settings and identify the estimation bias in the measured signals. The measurements are compared with the readings taken with an FDA-approved contact-based sensor for the same challenging dataset. The factors causing pulse rate variability with video-based methods are identified and compared with the factors causing pulse rate variability with the FDA-approved contact-based sensor. This work investigates the factors impacting pulse rate estimation of rPPG methods, as opposed to factors impacting PPG methods discussed by Bent et al.^11^.

Table 1 summarizes the aforementioned studies and focuses on the properties of the dataset used for evaluation, such as subject demographic diversity, presence of a laboratory setting, and the inclusion of an outside-the-lab study. This study investigates the performance of rPPG methods on data captured in the environment that is representative of our diverse subjects and hence, includes features and artifacts not typically explored in other works. Knowledge gained from this work will help inform researchers of the opportunities and challenges in deploying rPPG methods in diverse, real-world conditions.

**Table 1:** Summary of contact-based PPG and rPPG methods for pulse rate monitoring.

Authors	Year	Contact sensor-based on light reflection	Noncontact video-based	Subject demographic diversity	Lab control environment	Outside-the-lab study
Yang et al.^15^	1998	✓				
Rhee et al.^16^	2001	✓				
Renevey et al.^17^	2001	✓			✓	
Celka et al.^20^	2004	✓			✓	
Mendelson and Pujary^18^	2006	✓			✓	
Wang et al.^19^	2007	✓			✓	
Verkruysee et al.^27^	2008		✓		✓	
Poh et al.^29^	2010		✓		✓	
Poh et al.^21^	2010	✓			✓	
Lewandowska et al.^28^	2011		✓		✓	
Hao-Yu Wu et al.^30^	2012		✓		✓	
Tamura^23^	2014	✓			✓	
De Haan and Van Leest^31^	2014		✓	✓	✓	
Tautan ^24^	2015	✓			✓	
Wang et al. (POS)^32^	2016		✓	✓	✓	
Wang et al. (SSR)^33^	2017		✓	✓	✓	
Castaneda^25^	2018	✓				
Prakash and Tucker (BKF)^34^	2018		✓	✓	✓	
Chen and McDuff (DeepPhys)^36^	2018		✓		✓	
Niu et al. (Synrhythm)^37^	2018		✓		✓	
Speltik et al. (HR-CNN)^38^	2018		✓		✓	
Pilz (Spherical Mean)^35^	2019		✓		✓	
Zhang^26^	2019	✓			✓	
Yu et al. (PhysNet)^39^	2019		✓		✓	
Bent et al. ^11^	2020	✓		✓	✓	
Current study	2021	✓	✓	✓		✓

## Results

### Data collection

**Fig. 2:**
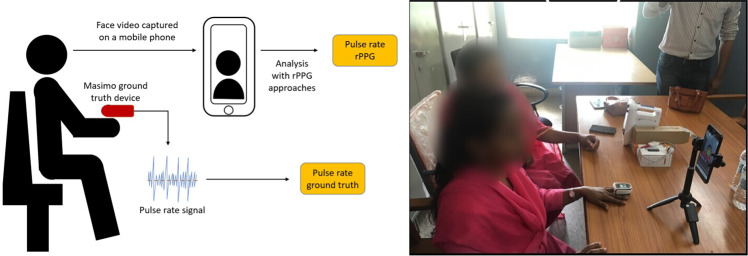
Experimental setup used for data collection. The graphical representation of the data collection process for rPPG approaches (mobile phone video) and the contact-based ground truth PPG (Masimo device) is shown in the left figure. The right figure depicts the actual data collection process showing the positioning of the ground truth Masimo device on the left hand and the subject facing the mobile phone camera in an upright position (faces blurred to maintain anonymity).

We collected the facial videos and ground truth pulse rate of 140 subjects (44 from India and 96 from Sierra Leone). The data were collected on different days to minimize random errors. The size of the dataset reported in this study is larger than some of the previous work, such as Bent et al.^11^. While their dataset reports equal distribution of individuals representing all skin tones, the demographics (with respect to country of origin) of their subjects are not reported. We developed the following protocol for data collection. For each subject, (1) ensure the subject is seated indoors and at rest, while facing the front camera of a mobile phone, (2) capture a facial video of the subject for a minimum of 120 s, (3) simultaneously collect the ground truth pulse rate of the subject using a contact-based pulse oximeter, (4) fill out a general survey giving details of nationality, gender, and age (Fig. 2). The duration of 120 s for video collection was decided based on the minimum length of video required for each rPPG algorithm. Though existing rPPG implementations use 60 s of video^52^, studies have shown that longer videos provide better estimates of pulse rate^53^. The 120 s duration was also found to capture sufficient subject motion and ambient lighting changes. All the subjects were completely rested before their pulse rate was recorded. The facial videos were captured using a Samsung J7 smartphone that captures 25 frames per second (f.p.s.) on average in ambient lighting conditions. The default smartphone camera settings, such as exposure time, are used for video capture, to test existing rPPG algorithms with a video provided by a typical smartphone camera. Since the default camera settings were used for video collection, the effect of camera settings on the accuracy of pulse rate determination is not a part of this study. The ground truth pulse rate was captured using the MightySat Rx P/N 9709 Masimo finger pulse oximeter (MightySat Rx P/N 9709 Masimo finger pulse-oximeter documentation). The study was conducted with the approval of and in accordance with the relevant guidelines and regulations of The Pennsylvania State University’s Institutional Review Board. Prior to the conduct of the study, informed consent was obtained from all participants for participation in the study. Only human subjects of age 18 or above were included in the study.

In the following section, we conduct statistical analyses between the pulse rate estimated by rPPG approaches and the ground truth pulse rate values. Since our statistical analyses involve between population comparison, we randomly sample 44 videos from the Sierra Leone dataset. The mean age of subjects from the Sierra Leone dataset after sampling is 45.0 years, for 22 female and 22 male subjects. The mean age of subjects from the India dataset is 37.0 years, for 25 female subjects and 19 male subjects. The overall mean age of subjects from both the countries is 41.9 years.

In this study, we consider the following baseline rPPG approaches: CHROM (2014), POS (2016), BKF (2018), DeepPhys (2018), and Spherical Mean (2019) for the evaluation against the ground truth, which is the Masimo pulse oximeter. The Masimo device is an FDA-approved transmittance type finger pulse oximeter that measures pulse rate by monitoring changes in the light passing through the finger tip^54^. We determine the pulse rate estimation biases in rPPG technqiues across diverse demographics to create a baseline evaluation procedure for rPPG methods. The demographic factors considered in this study are gender, country, and skin tone. We determine the pulse rate estimation bias in each rPPG approach and for all factors considered. Results of all hypothesis tests described in “Methods” section are discussed, and the factors causing statistically significant differences are analyzed.

### Analysis of the FDA-approved Masimo ground truth device

Performing the two-sample Kolmogorov–Smirnov (KS) test for the hypothesis described in the section “Tests across populations for the FDA-approved Masimo ground truth device” for the contact-based sensor, we fail to reject the null hypothesis with a *p* value of 0.94 (*α* = 0.05). Hence, there is no statistically significant difference in the mean pulse rate distributions between India and Sierra Leone.

We perform a two-sample KS test for each hypothesis test described in the section “Tests within populations for the FDA-approved Masimo ground truth device”, with a significance level of *α* = 0.0375 (as calculated in the section “Tests within populations for the FDA-approved Masimo ground truth device”).

For tests between genders in each country, we obtain *p* values of 0.05 and 0.39, respectively. This shows that there is no statistically significant difference in the mean pulse rate between subjects of different genders within the same country. For the tests comparing subjects of the same gender, but from different countries, we obtain a *p* value of 0.63 for female subjects and 0.05 for male subjects. The *p* values obtained fail to reject the null hypothesis and show that there is no statistically significant difference in the mean pulse rate for subjects of the same gender, but from different countries. The differences in the *p* values between countries and between genders could be attributed to factors, such as differences in diet or activity level, physiological variation, or even cultural distinctions, which cannot be tested with the collected data. Table 2 summarizes the findings.

**Table 2:** KS test—*p* value comparison with respect to gender.

Test	*p V*alues for Masimo
India (female, male)	0.05
Sierra Leone (female, male)	0.39
Female (India, Sierra Leone)	0.63
Male (India, Sierra Leone)	0.05

### Analysis of rPPG approaches

The results of the KS test conducted for each rPPG algorithm for complete samples from India and Sierra Leone are tabulated in Table 3.

**Table 3:** Results (*p* values) for hypothesis tests conducted across subjects of India and Sierra Leone for different rPPG approaches (test 15), compared to Masimo.

Algorithm	Type	(India, Sierra Leone)
CHROM	Non deep-learning rPPG	0.21
POS	Non deep-learning rPPG	0.64
BKF	Non deep-learning rPPG	0.47
Spherical Mean	Non deep-learning rPPG	0.13
DeepPhys	Deep-learning rPPG	0.08
Masimo	Ground truth PPG	0.94

The Masimo hypothesis testing shows a *p* value of 0.94, failing to reject the null hypothesis. This shows that the mean pulse rate distributions from India and Sierra Leone do not show a statistically significant difference with the contact-based sensor. We test the same hypothesis between India and Sierra Leone, using the results from the rPPG methods.

The BKF method shows a *p* value of 0.47, which exceeds the significance level for the hypothesis test, failing to reject the null hypothesis. This shows that the BKF approach does not show a significant difference in pulse rate between India and Sierra Leone. As tabulated in Table 3, the other conventional non deep-learning methods show *p* values exceeding the hypothesis test significance level, failing to reject the null hypothesis. The deep-learning-based DeepPhys network also shows a *p* value of 0.08 for the same hypothesis test, failing to reject the null hypothesis. This result shows that none of the rPPG methods show a statistically significant difference in mean pulse rate distributions between India and Sierra Leone, consistent with the findings of the Masimo ground truth device. This suggests that the performance of rPPG approaches could be comparable to the performance of the Masimo device on our dataset. In order to determine the level of agreement of pulse rate predicted by rPPG methods with the pulse rate shown by the Masimo device, we perform Bland Altman analysis between rPPG predictions and the Masimo device readings for all videos.

**Fig. 3:**
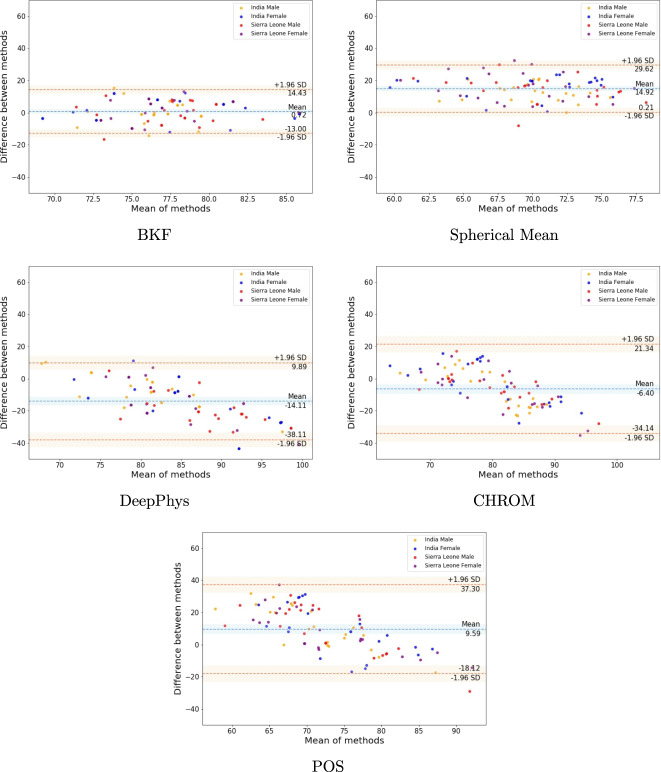
Bland Altman plots for rPPG approaches compared to the Masimo. The plots show the agreement of the selected rPPG methods (BKF, Spherical Mean, DeepPhys, CHROM and POS) with the Masimo device readings for male subjects from India (yellow), female subjects from India (blue), male subjects from Sierra Leone (red) and female subjects from Sierra Leone (purple). Error bars and 95% confidence intervals are marked (in b.p.m.).

The Bland Altman plots shown in Fig. 3 comparing predictions by each rPPG approach with the FDA-approved Masimo readings show points scattered above and below zero mean error for CHROM, POS, and BKF, suggesting that there is no consistent bias for these methods with the Masimo. The Spherical Mean approach shows most points lying completely above zero mean error and the DeepPhys approach shows most points completely below zero mean error, indicating the presence of a consistent bias with the Masimo. This shows that the Spherical Mean approach predicts a lower pulse rate than ground truth, while DeepPhys predicts a higher pulse rate than ground truth. CHROM and POS underpredict lower heart rate values and overpredict higher heart rate values. The overall mean error of rPPG approaches with the Masimo is 0.94 b.p.m. (Std. = 11 b.p.m.). We observe that rPPG methods, though accurate to within 1 b.p.m., (compared to Masimo readings) show a standard deviation of 11 b.p.m.. This is in contrast to the original studies introducing these rPPG methods, which reported pulse rate variabilities of 3–4 b.p.m.^31,32,34–36^ with ground truth. This difference in variability could be attributed to reasons, such as the difference in the selected ground truth device or the use of a diverse, challenging dataset for evaluation in this work. The devices used for ground truth pulse rate for the original rPPG studies are CMS50E pulse oximeter (2 b.p.m. error) for CHROM and Spherical Mean, ECG (no error) for POS, and DeepPhys and Polar H7 Bluetooth monitoring system for BKF. The measurement error rates of these ground truth devices are different from the measurement error rate of the Masimo device used for this study. Though ECG is considered the gold standard in estimating the pulse rate, it is not suitable to provide the ground truth for an outside-the-lab environment (e.g., the dataset collected in this study) due to the difficulty in installing the equipment. The source of variability for rPPG approaches with respect to the Masimo can also be attributed to the features present in our dataset, such as environmental lighting changes, head movements, and also facial marks (“bindi”) and headgear (hats and turbans). Features, such as changes in lighting and head movement, cause incorrect identification of the face in the video, leading to an incorrect pulse rate prediction. The presence of headgear or facial marks, such as “bindi”, causes occlusion of the face region of interest (generally the forehead and cheeks) and leads to erroneous estimation of pulse rate. The Bland Altman statistics for each rPPG algorithm are tabulated in Table 4.

**Table 4:** Bland Altman error statistics of rPPG methods compared to the Masimo (in b.p.m.).

Algorithm	Mean error	Std.
CHROM	−6.40	14.15
POS	9.59	14.14
BKF	0.72	6.99
Spherical Mean	14.92	7.50
DeepPhys	−14.11	12.24
Mean	0.94	11.00

We examined the videos deviating by two standard deviations from the mean to identify anomalies causing the high error with respect to the mean. We found nine such outlier videos containing one or more of the following anomalies, (1) excess or rapid head or camera movement, (2) poor or rapidly changing lighting conditions, and (3) the presence of an additional person in the frame. The aforementioned anomalies cause errors in face tracking resulting in incorrect identification of skin pixels and erroneous prediction of pulse rate. The camera tripod was subjected to movement in cases requiring adjustment to improve ambient lighting conditions or to ensure proper positioning of the face in the video frame. Since the data collection was performed in the subject’s natural setting, the background consisted of ambient lighting variations and people moving into the video frame. Since the selected rPPG methods estimate the pulse rate using all the identified regions of skin, the detection of a second person introduces noise in the estimation of the subject’s pulse rate. Table 5 details the types of anomalies found in the outlier videos. Conducting the KS test for each rPPG algorithm after removing the outlier videos, we obtain the *p* values tabulated in Table 6. For the rPPG methods, the *p* values obtained fail to reject the null hypothesis and show that there is no statistically significant difference in the mean pulse rate distributions of India and Sierra Leone, after removing the outlier videos. In addition, the *p* values obtained for the Masimo device also show that there is no statistically significant difference in the mean pulse rate distributions of India and Sierra Leone after removing the outlier videos. Performing the Bland Altman analysis after removing the nine outlier videos, we observe a reduction in the average standard deviation (11–10.05 b.p.m.; Table 7) of rPPG pulse rate predictions compared to the Masimo readings. From the analysis of the outlier videos, we define minimal head/camera movement, proper face lighting, and the presence of only one person in the video frame to be conditions for optimal performance of rPPG approaches in an outside-the-lab setting. Also, a larger percentage of outlier videos were found to be from Sierra Leone subjects than Indian subjects, leading us to hypothesize that differences in skin tone could impact rPPG approaches. The skin tone analysis is covered in detail in the sections “Variation of skin tone across populations” and “Impact of variation in skin tone on pulse rate estimation bias”.

**Table 5:** Frequency of anomalies in outlier videos (each video can have more than one anomaly).

	Rapid head/camera movement	Changing lighting/poor lighting	Presence of additional person
Video 1	✓		
Video 2	✓	✓	
Video 3	✓		✓
Video 4	✓		
Video 5			✓
Video 6	✓		
Video 7	✓		
Video 8		✓	
Video 9	✓	✓

**Table 6:** Results (*p* values) for hypothesis tests conducted across subjects of India and Sierra Leone for different rPPG approaches (test 15), compared to Masimo, after the removal of the outlier videos.

Algorithm	Type	(India, Sierra Leone)
CHROM	Non deep-learning rPPG	0.27
POS	Non deep-learning rPPG	0.27
BKF	Non deep-learning rPPG	0.40
Spherical Mean	Non deep-learning rPPG	0.40
DeepPhys	Deep-learning rPPG	0.05
Masimo	Ground truth PPG	0.77

**Table 7:** Bland Altman error statistics of rPPG methods compared to the Masimo after the removal of outlier videos (in b.p.m.).

Algorithm	Mean error	Std.
CHROM	−5.10	11.78
POS	9.12	14.43
BKF	1.03	6.32
Spherical Mean	14.95	6.53
DeepPhys	−14.41	11.17
Mean	1.12	10.05

The results (*p* values) of the KS test conducted for each rPPG algorithm for subjects of different genders within populations are tabulated in Table 8.

**Table 8:** Gender analysis within populations for rPPG methods showing *p* values for hypothesis tests defined in the section “Tests within populations for rPPG methods with respect to gender”.

Algorithm	India (female, male)	Sierra Leone (female, male)	Female (India, Sierra Leone)	Male (India, Sierra Leone)
CHROM	0.63	0.22	0.22	0.39
POS	0.39	0.99	0.63	0.11
BKF	0.05	0.22	0.05	0.05
Spherical Mean	0.63	0.63	0.39	0.11
DeepPhys	0.11	0.11	0.87	0.05
Masimo	0.05	0.39	0.63	0.05

The *p* values obtained for each rPPG method (Table 8) fail to reject the null hypothesis described in the section “Tests within populations for rPPG methods with respect to gender”, showing that there is no statistically significant difference for any rPPG method between the pulse rate distributions of subjects of different genders in the same country or subjects of the same gender, but in different countries. This result is consistent with the results of hypothesis tests of the section “Tests within populations for the FDA-approved Masimo ground truth device”, where the tests conducted on the Masimo device readings showed no statistically significant difference for subjects of different genders in the same country or subjects of the same gender, but in different countries. Conducting the hypothesis tests within populations (section “Tests within populations for rPPG methods with respect to gender”) after removing the outlier videos, we obtain the *p* values tabulated in Table 9. The *p* values obtained after the removal of outlier videos show that there is no statistically significant difference for the rPPG methods between pulse rate distributions of subjects of different genders within the same country or subjects of the same gender in different countries. In addition, the *p* values obtained for the Masimo device also show that there is no statistically significant difference between pulse rate distributions of subjects of different genders within the same country or subjects of the same gender, but in different countries.

**Table 9:** Gender analysis within populations for rPPG methods showing *p* values for hypothesis tests defined in the section “Tests within populations for rPPG methods with respect to gender” after the removal of the outlier videos.

Algorithm	India (female, male)	Sierra Leone (female, male)	Female (India, Sierra Leone)	Male (India, Sierra Leone)
CHROM	0.57	0.18	0.57	0.34
POS	0.18	0.83	0.57	0.34
BKF	0.18	0.57	0.18	0.08
Spherical Mean	0.98	0.98	0.57	0.57
DeepPhys	0.18	0.34	0.83	0.08
Masimo	0.04	0.34	0.18	0.34

This result combined with the result in the section “Analysis of the FDA-approved masimo ground truth device” shows that the pulse rate measured by rPPG methods does not show a significant estimation bias across or within countries and genders. The pulse rate measured by the FDA-approved sensor also shows no significant estimation bias across or within countries and genders. The removal of outlier videos does not reveal any statistically significant difference in the mean pulse rate across, or within countries and genders (Tables 6 and 9), but reduces the variability in the pulse rate measured with rPPG approaches (Tables 4 and 7). The performance of rPPG methods is comparable to that of the FDA-approved sensor, as observed in the Bland Altman plots, providing validity to our hypothesis that rPPG methods are sufficiently robust in challenging conditions.

### Variation of skin tone across populations

**Fig. 4:**
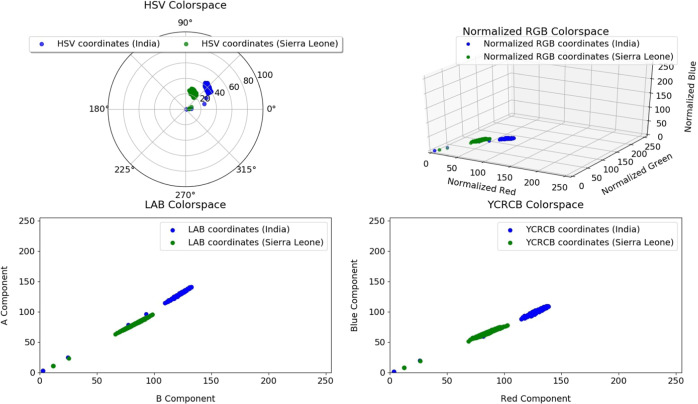
Color space visualization of skin pixels from Sierra Leone and India. The sub-plots show the variation of skin pixel intensities of subjects from India (blue) and Sierra Leone (green) in the HSV Color space (top left), the Normalized RGB Color space (top right), the LAB Color space (bottom left) and the YCrCb Color space (bottom right).

We analyze the temporal skin tone variation between Indian and Sierra Leone subjects to identify the impact of a difference in skin tone on the pulse rate estimation bias. Figure 4 illustrates a scatter plot of various chroma components of subjects from both populations. Following color space normalization and transformations, we now perform a between population skin tone analysis by keeping color as a response and varying population (country) and gender.

Let *f* denote a color space mapping function as follows, $f:{\mathcal{C}}\,\mapsto \,{{\mathcal{C}}}_{i}^{\prime}$, where ${{\mathcal{C}}}_{i}^{\prime}$ denotes the transformed color space *i* such that $i\,\in \,{\mathcal{S}}$ and ${\mathcal{S}}=[\rm{HSV,LAB,YCrCb}]$. We formally state and test the following hypotheses on each element of ${{\mathcal{C}}}_{i}^{\prime}$ for all color spaces considered, with a significance level of *α* = 0.05 from power analysis (effect size = 0.5 (Cohen’s *d*), no. of samples = 88, power = 0.8).

1 $${H}_{0}:P({{\mathcal{C}}}_{i}^{\prime}(\rm{India}))=P({{\mathcal{C}}}_{i}^{\prime}({\rm{Sierra}\;{\rm{Leone}}}))$$

2 $${H}_{a}:P({{\mathcal{C}}}_{i}^{\prime}(\rm{India}))\,\ne\, P({{\mathcal{C}}}_{i}^{\prime}({\rm{Sierra}}\;{\rm{Leone}}))$$

We perform a two-sample KS test between the Hue ${{\mathcal{C}}}_{i}^{\prime}(\rm{India})$ and Hue ${{\mathcal{C}}}_{i}^{\prime}({{\rm{Sierra}}\;{\rm{Leone}}})$ pixel distributions, and we reject the null hypothesis with *p* values tabulated in Table 10.

**Table 10:** KS test—*p* value comparison between population samples (gray shading highlights a statistically significant result).

Color Component (color space)	(India, Sierra Leone)
Normalized red (RGB)	0.00
Normalized green (RGB)	0.01
Normalized blue (RGB)	0.03
Hue (HSV)	0.00
A component (LAB)	0.02
B Component (LAB)	0.00
Component red (YCrCb)	0.00
Component blue (YCrCb)	0.03

Through hypothesis testing, we conclude that there is a statistically significant difference between skin tone samples from two ethnically and geographically divided populations. Figure 4 shows the large difference in the mean skin tone of subjects from India and Sierra Leone. The mean pulse rate distributions however do not show a statistically significant difference between countries (section “Analysis of rPPG approaches”) for rPPG methods, suggesting that skin tone may not be a critical factor impacting the bias in estimating pulse rate.

## Discussion

In this study, we present an analysis of pulse rate estimation bias of rPPG approaches across subjects with diverse demographic backgrounds in outside-the-lab conditions. A key achievement is the collection of a demographically diverse dataset, collected in outside-the-lab settings. The collected dataset consists of videos of subjects with unique facial and cultural features under dynamic lighting conditions, making it different from currently available face video datasets. Also, the subjects in the dataset collected belong to demographics (country and skin tone) typically not represented in existing face video datasets. We hypothesize that state-of-the-art rPPG pulse rate estimation methods should not differentiate between pulse rate distributions of subjects from different countries or between pulse rate distributions of subjects belonging to different genders from our dataset. To this effect, we tested five different rPPG pulse rate estimation methods on our dataset, using an FDA-approved contact-based device as the ground truth. Performing hypothesis testing, we found that the selected rPPG approaches do not make a distinction between subjects of different countries or different genders. Interestingly, this result is in accordance with the results of hypothesis testing between countries and genders for the FDA-approved ground truth sensor, showing that rPPG methods perform on a scale comparable to the FDA-approved sensor. To determine the exact level of error between pulse rates predicted by rPPG approaches and the ground truth, we conducted a Bland Altman analysis comparing pulse rate predictions of each rPPG approach with the ground truth sensor readings. Surprisingly, we found that though the mean pulse rate error of rPPG predictions with the ground truth readings was within acceptable limits, the variability in error was higher than the values reported in the studies introducing the rPPG methods. We find that the use of a different ground truth sensor in our study may partly contribute to the higher variability. Analyzing the videos causing the highest error (2 Std. from mean), we observe that these videos show excess subject movement and rapid lighting changes. Also, some of these videos contain more than one person in the frame. Removing these videos, we conducted the Bland Altman analysis and hypothesis testing once again comparing each rPPG approach with the ground truth sensor and found that the variability in pulse rate error decreased, though the pulse rate distributions do not show a statistically significant difference between countries or genders. We thus postulate that the presence of subject or environmental disturbances in videos causes higher pulse rate measurement error with rPPG approaches. The key finding from this study is that rPPG approaches generalize well to datasets with subject features not typically seen (such as different countries, darker skin tones, or facial marks/cultural artifacts) if the subject motion or environmental disturbance is maintained within acceptable limits. The quantification of the acceptable limits for subject motion or environmental disturbance is part of future exploration. This work provides motivation for future studies to explore more diverse datasets or physiological signals other than the pulse rate to improve the generalizability of rPPG approaches.

## Methods

### Overview

**Fig. 5:**
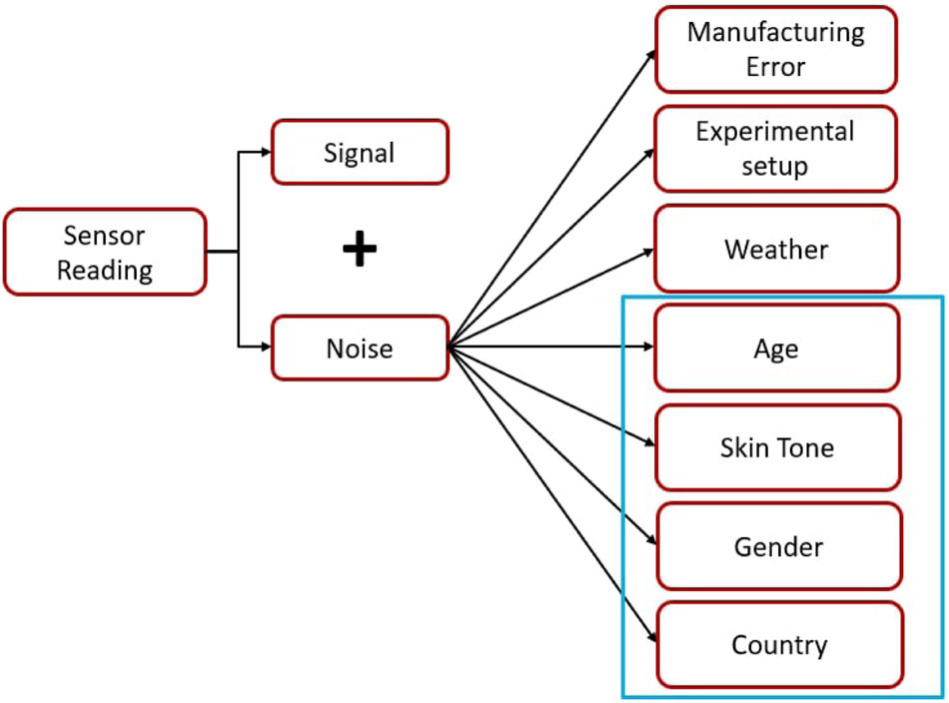
Sources of noise in the measured signal, highlighting the sources covered in this study. The variation of heart rate measured with rPPG methods across the demographic factors of age, skin tone, gender and country of origin, is analyzed using statistical hypothesis testing.

Figure 5 illustrates our data collection process. Each sensor reading consists of a main signal that is associated with a noise signal. The source of the noise signal is a combination of factors, such as conditions during measurement and bias due to subject demographics, as shown in Fig. 5. The manufacturing error for the Masimo device is modeled as a normal distribution with mean = 0 b.p.m. and standard deviation = 3 b.p.m., as specified in its official operating manual (Masimo Operating Manual). Since data collection in India and Sierra Leone followed the same experimental protocol, the setup error can be assumed to be minimal and hence can be neglected. The weather data of the location was collected, and the temperature (Climate of the World) was found to be within the Masimo device’s acceptable range specified by the manufacturer (Masimo Operating Manual), thus minimizing measurement error due to local weather changes. Hence, we neglect weather as a source of error in our study. To analyze the age distributions of the subjects from India and Sierra Leone, we design a hypothesis test as follows:

3 $${H}_{0}:{\rm{Age}}\;{\rm{(India)}={\rm{Age}}\;{\rm{(Sierra}}\,{\rm{Leone)}}}$$

4 $${H}_{\rm{a}}:{\rm{Age}\;{\rm{(India)}}\,\ne\, {\rm{Age}}\;{\rm{(Sierra}}\,{\rm{Leone)}}}$$

Using power analysis, we determine a significance level of *α* = 0.05 (effect size = 0.5 (Cohen’s *d* (ref. ^55^)), no. of samples = 88, power = 0.8, degrees of freedom = 1). We perform a two-sided KS test and we fail to reject the null hypothesis with a *p* value of 0.73. This shows that the age distributions of the two countries show no statistically significant difference between them. The similarity in the age distributions of India and Sierra Leone shows that any pulse rate bias between the subjects of the two countries cannot be attributed to differences in subject ages. In the following sections, we test the bias in rPPG approaches due to change in gender, country of origin, and skin tone.

### Contact-based sensor analysis across demographics

The FDA-approved contact-based sensor was tested for two factors—country and gender. Demographic groups on the basis of gender and country are defined as: Gender = [female, male] and Country = [India, Sierra Leone]. Hypothesis tests are designed to (i) analyze variations between populations and (ii) analyze variations within populations. The tests are designed to determine the variation of pulse rate measurement error across different groups. For larger demographic groups, (such as the entire population sample of each country) the significance level of *α* = 0.05 is computed based on power analysis (effect size = 0.5 (Cohen’s *d*), no. of samples = 88, power = 0.8, degrees of freedom = 1). For smaller demographic groups, (such as female subjects) the significance level of *α* = 0.15 is determined, according to power analysis (effect size = 0.5 (Cohen’s *d*), no. of samples = 44, power = 0.8, degrees of freedom = 1).

### Tests across populations for the FDA-approved Masimo ground truth device

Hypothesis tests are defined to identify pulse rate variations between the sample populations of India and Sierra Leone. Let ${{\widehat{\rm{PR}}{({\rm{India}})}}}$ and ${\widehat{\rm{PR}}{({\rm{Sierra}}}}\,{\rm{Leone}})$ be the mean distributions of pulse rate for all subjects from India and Sierra Leone, respectively. The hypothesis is formally stated as,

5 $${H}_{0}:{{\widehat{\rm{PR}}{({\rm{India}})}}={{\widehat{\rm{PR}}}}({{\rm{Sierra}}}}\,{\rm{Leone}})$$

6 $${H}_{\rm{a}}:{{\widehat{\rm{PR}}{({\rm{India}})}}\,\ne\, {{\widehat{\rm{PR}}}}({{\rm{Sierra}}}}\,{\rm{Leone}})$$

where, *H*_0_ and *H*_a_ are the null and alternate hypotheses, respectively. We perform a two-sided KS test between the mean pulse rate distributions from India and Sierra Leone to determine if there exists a statistically significant difference between the two-sample populations.

### Tests within populations for the FDA-approved Masimo ground truth device

The presence of a statistically significant difference in mean pulse rate distributions within sample populations requires hypothesis testing with respect to gender. First, we test for variation in pulse rate between the two genders within each sample population, in order to determine if change in gender affects the pulse rate distribution. The hypothesis is formally stated as,

For sample population from India,

7 $${H}_{0}:{\widehat{\rm{PR}}({{{\rm{India}}}}_{\rm{{Female}}})={\widehat{\rm{PR}}}({\rm{India}}_{\rm{{Male}}}})$$

8 $${H}_{\rm{a}}:{\widehat{\rm{PR}}({{{\rm{India}}}}_{\rm{{Female}}})\,\ne\,{\widehat{\rm{PR}}}({\rm{India}}_{\rm{{Male}}}})$$

and for Sierra Leone,

9 $${H}_{0}:{\widehat{\rm{PR}}({\rm{Sierra}}\,{\rm{Leon}}_{\rm{{Female}}})={\widehat{\rm{PR}}}({\rm{Sierra}}\,{\rm{Leon}}_{\rm{{Male}}}})$$

10 $${H}_{\rm{a}}:{\widehat{\rm{PR}}({\rm{Sierra}}\,{\rm{Leon}}_{\rm{{Female}}})\,\ne\,{\widehat{\rm{PR}}}({\rm{Sierra}}\,{\rm{Leon}}_{\rm{{Male}}}})$$

where *H*_0_ and *H*_a_ are the null and alternate hypotheses, respectively.

Following the within population gender hypothesis testing, we compare the mean pulse rate distributions for subjects of the same gender, but from different sample populations. The tests are defined as follows,

11 $${H}_{0}:{\widehat{\rm{PR}}({\rm{India}}_{\rm{{Female}}})={\widehat{\rm{PR}}}({\rm{Sierra}}\,{\rm{Leon}}_{\rm{{Female}}}})$$

12 $${H}_{\rm{{a}}}:{\widehat{\rm{PR}}({\rm{India}}_{\rm{{Female}}})\,\ne\,{\widehat{\rm{PR}}}({\rm{Sierra}}\,{\rm{Leon}}_{\rm{{Female}}}})$$

13 $${H}_{0}:{\widehat{\rm{PR}}({\rm{India}}_{\rm{{Male}}})={\widehat{\rm{PR}}}({\rm{Sierra}}\,{\rm{Leon}}_{\rm{{Male}}}})$$

14 $${H}_{\rm{a}}:{\widehat{\rm{PR}}({\rm{India}}_{\rm{{Male}}})\,\ne\,{\widehat{\rm{PR}}}({\rm{Sierra}}\,{\rm{Leon}}_{\rm{{Male}}}})$$

where *H*_0_ and *H*_a_ are the null and alternate hypotheses, respectively. Using power analysis, for demographic groups with fewer samples, the calculated significance level is *α* = 0.15. Four hypothesis tests need to be conducted to investigate the effect of gender, warranting the use of multiple hypothesis testing theory^56^. Due to the small number of hypotheses (here, four), multiple hypothesis testing is incorporated by dividing the original significance level by the number of tests (Bonferroni Correction)^57^. The final Bonferroni-corrected significance level for testing the effect of gender is *α* = 0.15/4 = 0.0375.

### Analysis of rPPG approaches across demographics

**Fig. 6:**
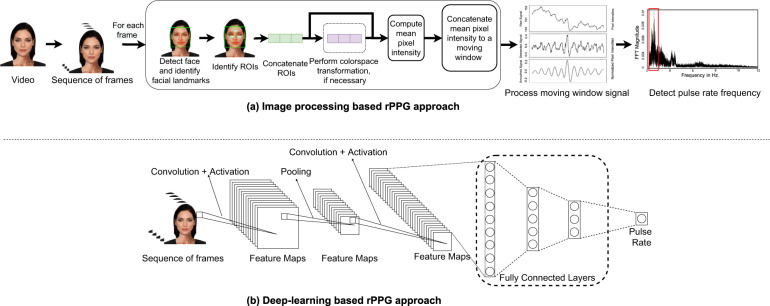
rPPG approaches to estimating pulse rate. Figure **a** (top) shows image processing-based detection of heart rate from a facial video, detailing the steps of face detection, skin segmentation, color space transformation and signal processing. Figure **b** (bottom) shows a deep convolutional neural network that predicts the heart rate using a facial video as the input.

Figure 6 presents a visual illustration of the workings of rPPG-based pulse rate estimation using image processing and deep-learning approaches. Both image processing and deep-learning methods use a sequence of frames as input to estimate the pulse rate. The image processing approach uses a multistep process for face detection, skin segmentation, and color space transformations, involving handcrafting of features, noise filtering, and signal processing as opposed to deep-learning approaches, where intermediate processing steps are learnt by the model.

Each rPPG method outputs a pulse rate reading for every 5 s of video. The pulse rate for the complete video is calculated by computing the mean of all the readings obtained over the duration of the video (120 s). Each rPPG algorithm also includes a video processing step, where color space transformations are performed to reduce the impact of bright external lights on the accuracy of pulse rate measurement. Hypothesis tests as described in the section “Tests across populations for the FDA-approved Masimo ground truth device” are performed across populations for each of the baseline rPPG approaches. The results are compared with the measurements of the Masimo pulse oximeter (averaged over 120 s) for each of the corresponding videos. The factors affecting the mean pulse rate distribution are identified and compared with the factors affecting the mean pulse rate captured by the Masimo device. The level of agreement between the two approaches provides a quantitative evaluation of the robustness of rPPG methods in measuring pulse rate of subjects across demographic and environmental conditions.

### Tests across populations for rPPG approaches

Hypothesis tests are defined to identify pulse rate variations in sample populations from India and Sierra Leone for each rPPG method. The presence of such variations would require further hypothesis tests with respect to gender, as described in the section “Tests within populations for the FDA-approved Masimo ground truth device”. The hypothesis for each rPPG approach is defined as,

15 $${H}_{0}:{{\widehat{\rm{PR}}{({\rm{India}})}}={{\widehat{\rm{PR}}}}({{\rm{Sierra}}}}\,{\rm{Leone}})$$

16 $${H}_{\rm{{a}}}:{{\widehat{\rm{PR}}{({\rm{India}})}}\,\ne\,{{\widehat{\rm{PR}}}}({{\rm{Sierra}}}}\,{\rm{Leone}})$$

where, ${{\widehat{\rm{PR}}{({\rm{India}})}}}$ and ${\widehat{\rm{PR}}{({\rm{Sierra}}}}\,{\rm{Leone}})$ are the mean pulse rate distributions of all subjects from India and Sierra Leone, respectively, measured using the rPPG approach. The significance level *α* is determined to be 0.05 using power analysis (effect size = 0.5 (Cohen’s *d*), no. of samples = 88, power = 0.8). Two-sided KS tests are performed to identify variations in mean pulse rate distributions between the sample populations from the two countries.

### Tests within populations for rPPG methods with respect to gender

Testing with respect to gender is required if the hypothesis test conducted for sample populations (section “Tests across populations for rPPG approaches”) shows a significant difference in mean pulse rate distributions. These hypothesis tests are designed as described in the section “Tests within populations for the FDA-approved Masimo ground truth device” for each video-based method. Using multiple hypothesis testing theory and applying Bonferroni’s correction, we set the significance level *α* to be 0.0375 for hypothesis tests analyzing the effect of gender as described in the section “Tests within populations for the FDA-approved Masimo ground truth device”.

### Impact of variation in skin tone on pulse rate estimation bias

We investigate the effect of skin tone on pulse rate measurement and analyze differences in skin tone between subjects from different demographic groups. PPG-based vital technologies measure variation in blood volume (pulse rate) by computing the change in pixel intensity values in a particular color space or a particular channel from a color space^58^. The total illumination from a region of interest, such as human skin can typically be captured as skin reflectance by an optical sensor, such as a camera^59^, which typically decomposes the reflected light using red (R), green (G), and blue (B) components of the RGB color space. However, the dichromatic reflection model suggests that light reflected from the skin consists of two components, namely a diffuse component and a specular component. Between these components, the light which gets reflected back from the skin after undergoing subsurface scattering is called the diffuse reflectance component^60^ and due to its interaction with skin contains more physiological information^61^.

The skin color of a face region of interest captured by a camera and averaged over all frames of the video is dependent on the relative contribution of specular and diffuse reflectance components, which again is dependent on the angles between camera, skin, and light source^53^. Hence, in order to compensate for these variations while preserving color information, we perform a per frame per channel RGB color space standardization and normalization on each element of the following tuple, which we consider as the default color space tuple:

17 $${\mathcal{C}}=\frac{(\frac{\rm{R}-{\mu }_{\rm{R}}}{{\sigma }_{\rm{R}}}),(\frac{\rm{G}-{\mu }_{\rm{G}}}{{\sigma }_{\rm{G}}}),(\frac{\rm{B}-{\mu }_{\rm{B}}}{{\sigma }_{\rm{B}}})}{\rm{R+G+B}}$$

where *μ*_(_._)_ and *σ*_(_._)_ denotes the mean and standard deviation of each element in the tuple (${\mathcal{C}}$) respectively. Following the normalization and standardization, the sum of the elements in ${\mathcal{C}}$ sum to 1. However, though the color space normalization is advantageous in that it compensates for unequal distribution of lighting, as well as change in color of lighting (other than white light)^53^, it does not however remove the specular components from the normalized R, G, and B channels. Hence, we further decompose ${\mathcal{C}}$ into chroma (color information) and luma (lighting intensity information) signals by exploring transforming to alternate color spaces, such as HSV (Hue-Saturation-Value), LAB (Luma component, A and B chroma components), and YCrCb (Luma component, blue-difference and red-difference chroma components) color spaces, which inherently represent pixel values in a decomposed form.

**Fig. 7:**
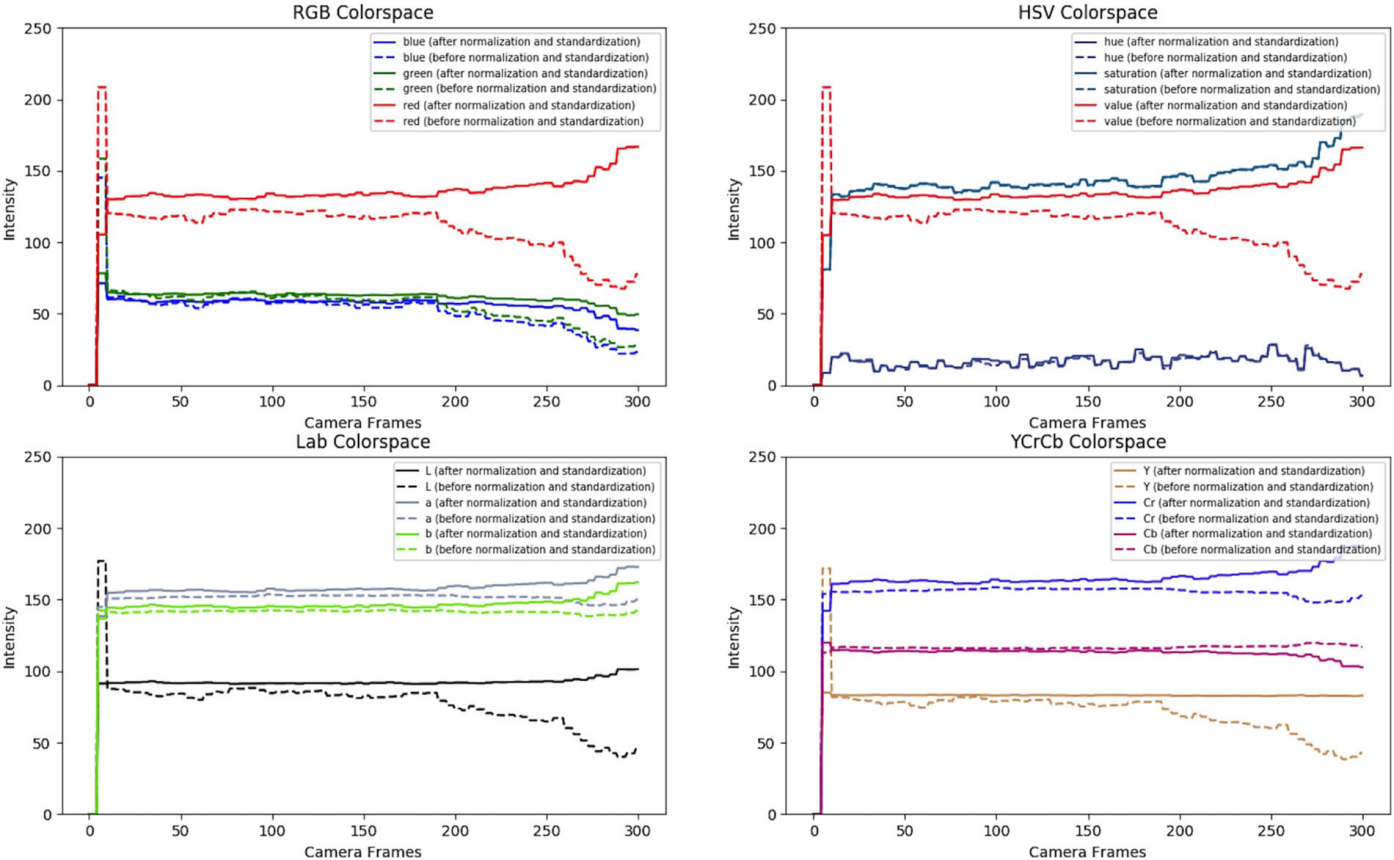
Comparison of 10 s time window pixel intensities in various color spaces after and before normalization for a random subject. Pixel intensities after normalization are denoted by solid lines and pixel intensities before normalization are denoted by dashed lines. Variation of pixel intensities in individual components of the RGB (top left), HSV (top right), LAB (bottom left) and YCrCb (bottom right) color spaces are depicted.

Figure 7 presents a time analysis of the varying pixel intensities in the RGB, HSV, YCrCb, and LAB color spaces before and after standardization and normalization for a randomly selected subject from our dataset. The time analysis reveals that color spaces that are able to seperate luminance information from chrominance information indeed preserve the chrominance information, and remain unaltered post standardization and normalization. This characteristic of HSV, LAB, and YCrCb color spaces make them suitable for estimating pulse rate in the presence of varying background lighting and brightness. While the variations between the normalized, standardized RGB pixel intensities, and the un-normalized RGB pixel intensities are fairly large, the reason for such variations is due to lighting intensity changes^53^. While Haan and Jeanne^53^ propose an empirical ratio for skin-tone standardization in the presence of unknown and colored lighting sources, such a standardization is primarily beneficial to the R, G, and B components of the RGB color space, as shown in the paper. Tsouri and Li^58^ on the other hand analyze the benefits of using alternative color spaces, such as HSV, HSI, CIE XYZ, etc. to estimate pulse rate and find that HSV and CIE XYZ are indeed better replacements to RGB color space for non-contact pulse rate estimation. Our analysis also shows that color preserving color spaces preserve chrominance information under non-white illumination and varying background lighting color and intensity. Chrominance preserving color spaces can be utilized by future rPPG algorithms for pulse rate estimation.

### Reporting summary

Further information on research design is available in the Nature Research Reporting Summary linked to this article.

## Supplementary information

Reporting Summary

## Data Availability

The dataset collected in this study is to be uploaded to https://github.com/ananyananda-dasari/bias-eval-rppg. To protect subject privacy, a frame-by-frame skin region of interest color space value dataset is to be uploaded instead of actual videos.

## Code availability

All code used for statistical analysis is open source with no restrictions and can be found on https://github.com/ananyananda-dasari/bias-eval-rppg. Code used for each rPPG algorithm is sourced from the respective public repositories.
